# FOXO are required for intervertebral disk homeostasis during aging and their deficiency promotes disk degeneration

**DOI:** 10.1111/acel.12800

**Published:** 2018-07-02

**Authors:** Oscar Alvarez‐Garcia, Tokio Matsuzaki, Merissa Olmer, Kohei Miyata, Sho Mokuda, Daisuke Sakai, Koichi Masuda, Hiroshi Asahara, Martin K. Lotz

**Affiliations:** ^1^ Department of Molecular Medicine The Scripps Research Institute La Jolla California; ^2^ Department of Orthopedic Surgery Tokai University School of Medicine Isehara‐shi Japan; ^3^ Department of Orthopedic Surgery University of California‐San Diego, Altman Clinical Translational Research Institute La Jolla California

**Keywords:** aging, autophagy, FOXO, intervertebral disk

## Abstract

Intervertebral disk (IVD) degeneration is a prevalent age‐associated musculoskeletal disorder and a major cause of chronic low back pain. Aging is the main risk factor for the disease, but the molecular mechanisms regulating IVD homeostasis during aging are unknown. The aim of this study was to investigate the function of FOXO, a family of transcription factors linked to aging and longevity, in IVD aging and age‐related degeneration. Conditional deletion of all FOXO isoforms (FOXO1, 3, and 4) in IVD using the Col2a1Cre and AcanCreER mouse resulted in spontaneous development of IVD degeneration that was driven by severe cell loss in the nucleus pulposus (NP) and cartilaginous endplates (EP). Conditional deletion of individual FOXO in mature mice showed that FOXO1 and FOXO3 are the dominant isoforms and have redundant functions in promoting IVD homeostasis. Gene expression analyses indicated impaired autophagy and reduced antioxidant defenses in the NP of FOXO‐deficient IVD. In primary human NP cells, FOXO directly regulated autophagy and adaptation to hypoxia and promoted resistance to oxidative and inflammatory stress. Our findings demonstrate that FOXO are critical regulators of IVD homeostasis during aging and suggest that maintaining or restoring FOXO expression can be a therapeutic strategy to promote healthy IVD aging and delay the onset of IVD degeneration.

## 1 INTRODUCTION

Intervertebral disk (IVD) degeneration (IDD) and associated changes in other spine tissues cause back pain and severe mobility limitations. This represents the most common musculoskeletal disorder and a major cause of disability (Dagenais, Caro, & Haldeman, 2008; Manchikanti, Singh, Falco, Benyamin, & Hirsch, 2014). The principal underlying causes of IDD are changes in cell numbers and phenotype and compromised biomechanical function in the IVD (Deyo & Weinstein, 2001). Aging is the major risk factor for IDD (Miller, Schmatz, & Schultz, 1988), and previous studies achieved substantial progress in describing age‐related changes in all IVD tissues, namely nucleus pulposus (NP), annulus fibrosus (AF), and endplates (EP) (Anderson & Tannoury, 2005; Boos et al., 2002; Kadow, Sowa, Vo, & Kang, 2015). Unlike any other connective tissues, age‐related changes in the IVD start early in life and involve a reduction in viable cells, particularly in the NP, and changes at the cellular and molecular level result in structural and functional impairment of the ECM, eventually leading to biomechanical failure and degeneration (Anderson & Tannoury, 2005; Kepler, Ponnappan, Tannoury, Risbud, & Anderson, 2013; Sivan, Wachtel, & Roughley, 2014; Vergroesen et al., 2015). However, the initiating molecular events that drive IVD aging remain poorly understood.

FOXO proteins are family of transcription factors that have important functions in development, aging, and longevity (Kahn, 2015; Martins, Lithgow, & Link, 2016). FOXO1, FOXO3, and FOXO4 are ubiquitously expressed, whereas FOXO6 expression is mainly detected in the brain (Eijkelenboom & Burgering, 2013). FOXO function in controlling cellular homeostasis and maintaining stem/progenitor cell populations, processes that are critical in aging and in determining lifespan (Eijkelenboom & Burgering, 2013; Webb & Brunet, 2014). Recent evidence supports that dysregulation of FOXO expression or activity contributes to the pathogenesis of age‐related diseases (Ambrogini et al., 2010; Sandri et al., 2004), including osteoarthritis (Akasaki et al., 2014; Matsuzaki et al., 2018).

We recently reported that expression of FOXO1 and FOXO3 in IVD is reduced during aging and degeneration in humans and mice (Alvarez‐Garcia, Matsuzaki, Olmer, Masuda, & Lotz, 2017). However, the function of FOXO in IVD is unknown. In this study, we used in vivo and in vitro approaches to investigate the role of this important family of transcription factors in IVD biology and pathogenesis of IDD.

## 2 RESULTS

### 2.1 FOXO deficiency impairs postnatal IVD maturation and homeostasis

To model and study the consequences of the reduced expression of FOXO in NP, AF, and EP of degenerated IVD (Alvarez‐Garcia, Matsuzaki, Olmer, Masuda, et al., 2017), we generated mice with deletion of FOXO1, 3, and 4 in IVD tissues under the *Col2a1* promoter (Col2a1‐Cre^+/−^; Foxo1^fl/fl^; Foxo3^fl/fl^; Foxo4^fl/fl^, herein referred as to Col2a1Cre‐FOXO KO). Col2a1‐Cre^−/−^; Foxo1^fl/fl^; Foxo3^fl/fl^; and Foxo4^fl/fl^ littermates (Col2a1Cre^−/−^) were used as controls. FOXO deletion was confirmed by gene expression analysis in NP and AF of lumbar IVD from 2‐month‐old mice (Supporting information Figure S1a in Appendix S1).

**Figure 1:**
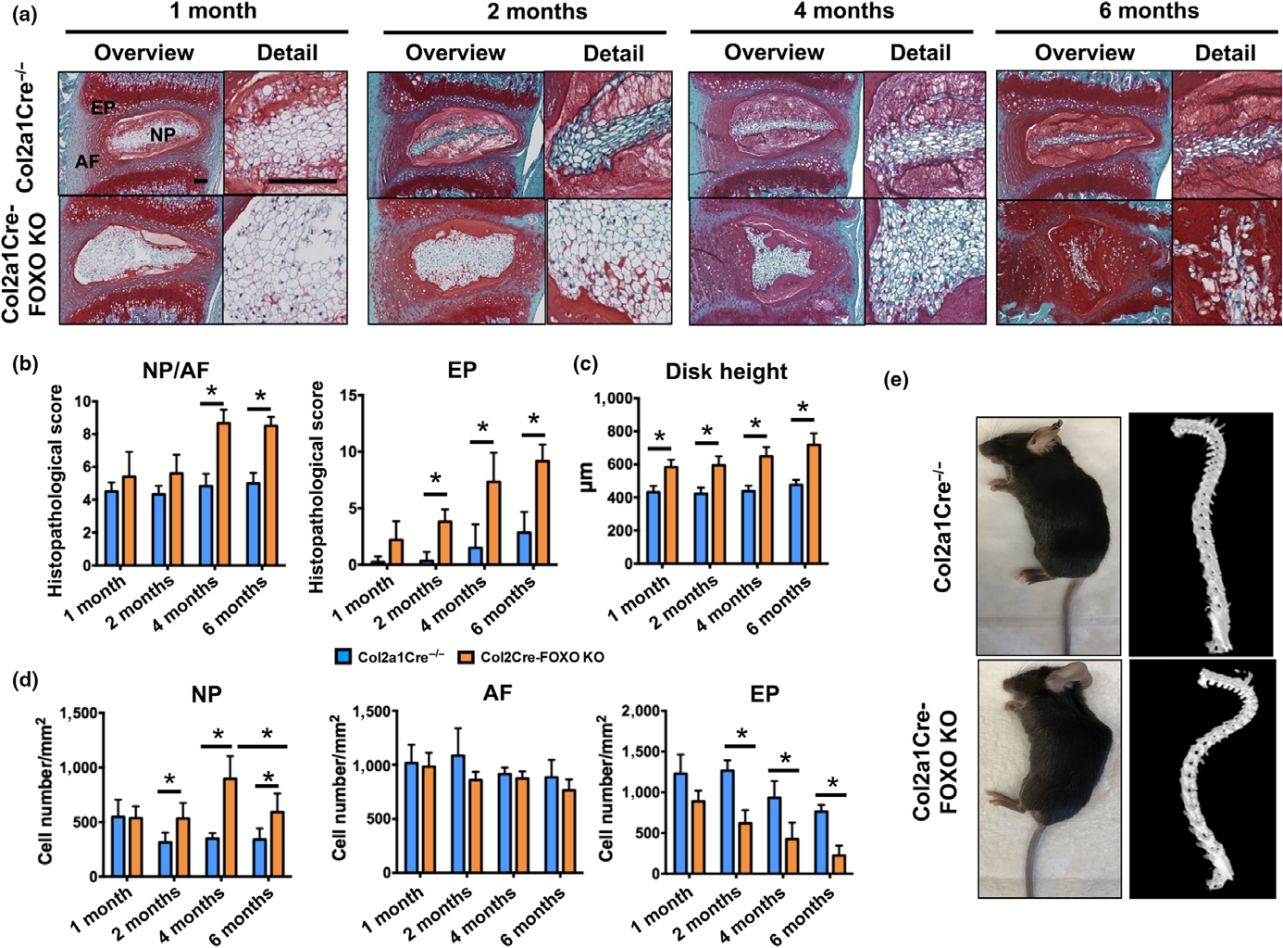
Impaired intervertebral disk maturation and spontaneous degeneration in mice with conditional deletion of FOXO. (a) Safranin O staining in lumbar intervertebral disk (IVD) samples isolated from Col2a1Cre^−/−^ and Col2a1Cre‐FOXO KO mice at 1, 2, 4, and 6 months of age (*n* = 6–8 mice per group). NP: nucleus pulposus; AF: annulus fibrosus; EP: endplate. Magnification bar = 100 µm. (b) Histopathological scores in the nucleus NP/AF and EP of lumbar IVD samples isolated from Col2a1Cre^−/−^ and Col2a1Cre‐FOXO KO mice at 1, 2, 4, and 6 months of age (*n* = 6–8 mice per group). (c) Measurement of disk height of lumbar IVD samples from Col2a1Cre^−/−^ and Col2a1Cre‐FOXO KO mice at 1, 2, 4, and 6 months of age (*n* = 6–8 mice per group). (d) Quantification of cellularity in the NP, AF, and EP of lumbar IVD samples isolated from Col2a1Cre^−/−^ and Col2a1Cre‐FOXO KO at 1, 2, 4, and 6 months of age (*n* = 6–8 mice per group). (e) Photograph (left) and µ‐CT image (right) of 6‐month‐old Col2a1Cre^−/−^ and Col2a1Cre‐FOXO TKO mice. Values shown are mean ± *SD*. Statistical comparisons were assessed by one‐way analysis of variance (ANOVA) followed by a post hoc Tukey's test. *p < 0.05

Col2a1Cre‐FOXO KO mice were viable at birth and had similar body size as Col2a1Cre^−/−^ littermates with no overt skeletal abnormalities. IVD from Col2a1Cre‐FOXO KO mice were indistinguishable from those in control mice at postnatal day 1 (P1) and 7 (P7) (Supporting information Figure S1b in Appendix S1). Starting at 1 month of age, lumbar IVD from Col2a1Cre‐FOXO KO mice exhibited a mild enlargement of the NP and a modest increase in disk height (Figure 1a–c). The increased disk height of mutant mice became more marked at 2, 4, and 6 months of age and was concomitant with significantly higher cellularity in the NP (Figure 1c–d). At 4 and 6 months of age, Col2a1Cre‐FOXO KO mice showed histological features of degeneration that included disruption of the NP/AF interface, disorganized AF lamellae with abundant hypertrophic cells in the inner AF, and cell loss and calcification of the EP (Figure 1b–d). In addition to the cell loss in the EP, there was a significant reduction in cellularity in the NP of Col2a1Cre‐FOXO KO mice at 6 months when compared to 4‐month‐old mice (Figure 1d). FOXO deficiency led to severe spine deformities with abnormal curvature of the spine and kyphosis in 6‐month‐old mice (Figure 1e). In addition, deletion of all FOXO isoforms resulted in abnormal cell organization in vertebral growth plate, increased vertebral diameter, increased trabeculae number, and trabecular thickness in subchondral bone at 4 and 6 months of age (Supporting information Figure S2 in Appendix S1).

To examine the function of each individual FOXO isoform in IVD growth and maintenance, we generated Col2a1Cre‐FOXO1 KO, Col2a1Cre‐FOXO3 KO, and Col2a1Cre‐FOXO4 KO mice. Col2a1Cre‐FOXO1 KO mice showed an increase in NP cellularity and cell hypertrophy in the inner AF (Supporting information Figure S3 in Appendix S1), suggesting that FOXO1 is the predominant isoform in the regulation of postnatal cell proliferation and differentiation. Reduced cellularity and mild degenerative changes were also observed in the EP of mice with FOXO1 deletion (Supporting information Figure S3 in Appendix S1). However, these changes were less severe than those in Col2a1Cre‐FOXO KO mice, indicating compensation by FOXO3 or FOXO4. On the other hand, Col2a1Cre‐FOXO3 KO and Col2a1Cre‐FOXO4 KO mice did not show any structural abnormalities in IVD up to 6 months of age. However, degenerative changes were observed in lumbar IVD from Col2a1Cre‐FOXO3 KO mice, but not Col2a1Cre‐FOXO4 KO, at 12 months (Supporting information Figure S4a in Appendix S1). These changes more severe at 18 months recapitulated histological hallmarks of age‐related disk degeneration such as decreased NP cellularity, decreased disk height, abnormal AF organization, and ossification of the EP and resulted in increased histopathological scores (Supporting information Figure S4b–d in Appendix S1). These results suggest that FOXO3 is dispensable for normal IVD postnatal development, likely due to compensation by FOXO1, but is essential for IVD homeostasis, and its deletion results in accelerated IDD during aging.

### 2.2 FOXO control IVD cell proliferation and maturation

**Figure 2:**
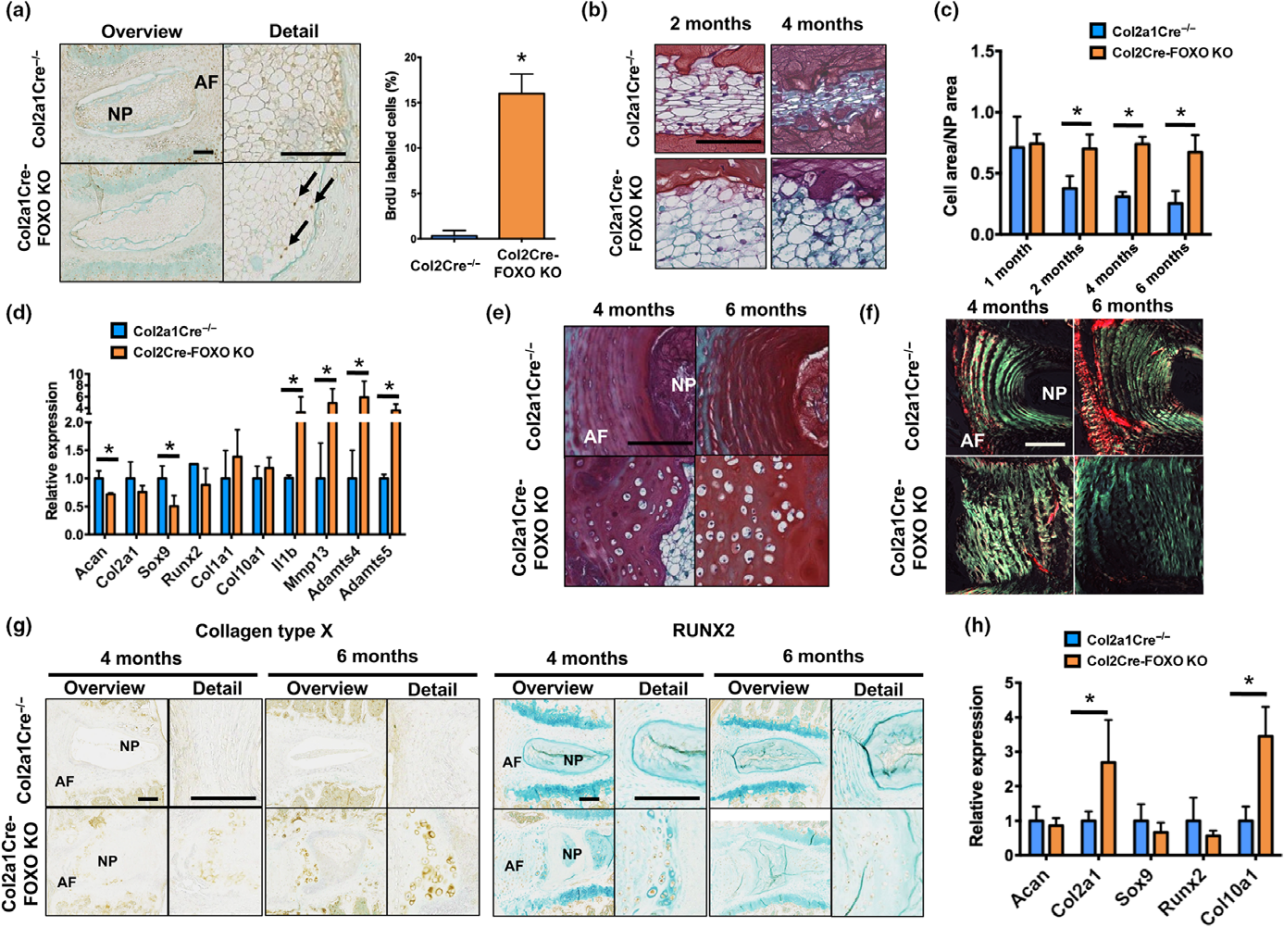
Regulation of intervertebral disk proliferation and differentiation by FOXO. (a) Immunohistochemical detection of 5‐bromo‐2'‐deoxyuridine (BrdU) in lumbar intervertebral disk (IVD) samples from Col2a1Cre^−/− ^and Col2a1Cre‐FOXO KO mice at 1 month of age (*n* = 3 mice per group) showing immunopositive cells (black arrows) in the nucleus pulposus (NP). AF: annulus fibrosus. Panel on the right shows quantification of BrdU‐positive cells in the NP. Magnification bar = 100 µm. (b) Safranin O staining in NP from lumbar IVD samples isolated from Col2a1Cre^−/−^ and Col2a1Cre‐FOXO KO mice at 2 and 4 months of age (*n* = 6–8 mice per group). Magnification bar = 50 µm. (c) Histomorphometric quantification of the ratio of the NP cell area to total NP area in IVD from Col2a1Cre^−/−^ and Col2a1Cre‐FOXO KO mice at 1, 2, 4, and 6 months of age (*n* = 6–8 mice per group). (d) Gene expression analysis of anabolic and catabolic genes in the NP of lumbar IVD samples from Col2a1Cre^−/− ^and Col2a1Cre‐FOXO KO at 4 months of age (*n* = 4 mice per group). (e) Safranin O staining in annulus fibrosus (AF) of lumbar IVD isolated from Col2a1Cre^−/− ^and Col2a1Cre‐FOXO KO mice at 4 and 6 months of age. Magnification bar = 100 µm. (f) Picrosirius red staining in lumbar IVD isolated from Col2a1Cre^−/−^ and Col2a1Cre‐FOXO KO mice at 4 and 6 months of age (*n* = 6 mice per group). Images were obtained under polarized light to show collagen birefringence. Magnification bar = 100 µm. (g) Collagen type X (left panel) and RUNX2 (right panel) immunohistochemistry in lumbar IVD from Col2a1Cre^−/−^ and Col2a1Cre‐FOXO KO mice at 4 and 6 months of age (*n* = 5 mice per group). Magnification bar = 100 µm. (h) Gene expression analysis in the AF of lumbar IVD samples from Col2a1Cre^−/−^ and Col2a1Cre‐FOXO KO at 4 months of age (*n* = 4 mice per group). Values shown are mean ± *SD*. Statistical comparisons were assessed by an unpaired, two‐tailed *t*‐test after testing for equal variance using an *F*‐test. Values are mean ± *SD*. *p < 0.05

To test whether the increased cellularity in the NP of mice lacking FOXO was due to changes in cell proliferation, 3‐week‐old Col2a1Cre‐FOXO KO and Col2a1Cre^−/− ^control mice were injected with 5‐bromo‐2'‐deoxyuridine (BrdU) intraperitoneally for five consecutive days. Quantification of BrdU labeling by immunohistochemistry showed a significant increase in proliferating cells in the NP of Col2a1Cre‐FOXO KO mice as compared to control mice (Figure 2a), whereas no BrdU‐positive cells were detected in the AF or EP of Col2a1Cre‐FOXO KO or control mice.

FOXO deletion in the IVD also resulted in histological changes suggestive of abnormal cell differentiation. In mice, the NP compartment is composed of a large cluster of cells in the center of the NP that is surrounded by a layer of proteoglycan‐rich matrix (Tam et al., 2017). During postnatal growth and maturation, cells within the NP undergo a morphologic shift from a round and vacuolated to a more flattened cell when skeletal maturity is reached at 4 months of age (Figure 2b). However, this shift did not occur in Col2a1Cre‐FOXO KO mice where large round cells could still be seen at 4 months of age (Figure 2b). In addition, whereas the cell area/NP area ratio was progressively reduced in control mice, no significant changes were seen in mutant mice (Figure 2c). Gene expression analysis of NP revealed that Col2a1Cre‐FOXO KO mice had significantly reduced expression of *Acan* and *Sox9* and increased expression of catabolic mediators (*Il1b*,* Mmp13*,* Adamts4*,* and* *Adamts5*) at 4 months of age (Figure 2d). Abnormal cell differentiation was also observed in the AF of Col2a1Cre‐FOXO KO mice as indicated by the presence of hypertrophic cells in the inner AF (Figure 2e), disorganized pattern of collagen fibers (Figure 2f), and increased collagen type X and RUNX2 expression in the inner AF (Figure 2g). Moreover, mRNA expression of collagen type II (*Col2a1*) and X (*Col10a1*) was increased in the AF of Col2a1Cre‐FOXO KO mice when compared with controls at 4 months of age (Figure 2h). Collectively, these data indicate that FOXO deletion in IVD tissues severely impairs normal postnatal IVD growth and maturation.

### 2.3 FOXO deletion in skeletally mature mice leads to spontaneous IVD degeneration

**Figure 3:**
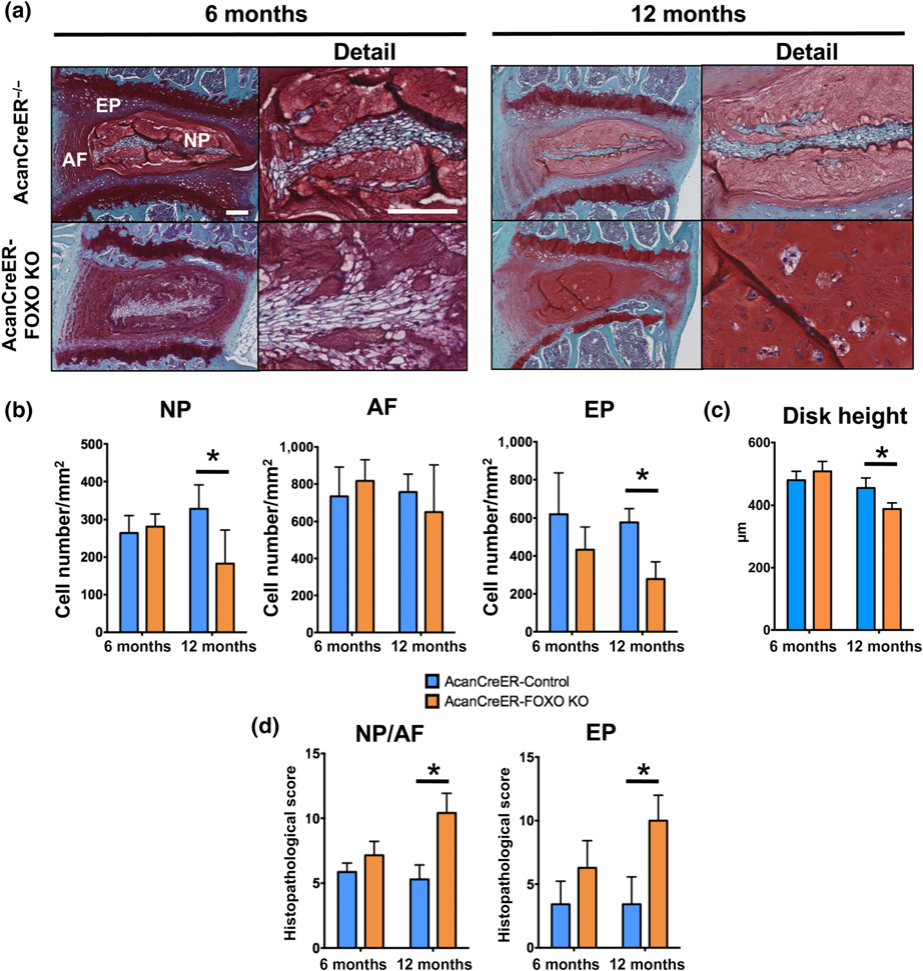
Spontaneous intervertebral disk degeneration in skeletally mature mice with conditional deletion of FOXO. (a) Safranin O staining in lumbar intervertebral disk (IVD) from AcanCreER^−/−^ and AcanCreER‐FOXO KO mice at 6 and 12 months of age (*n* = 6–8 mice per group). NP: nucleus pulposus; AF: annulus fibrosus; EP: endplate. Magnification bar = 100 µm. (b) Quantification of cellularity in NP, AF, and EP of lumbar IVD from AcanCreER^−/−^ and AcanCreER‐FOXO KO mice at 6 and 12 months of age (*n* = 6–8 mice per group). (c) Measurement of disk height of lumbar IVD samples AcanCreER^−/−^ and AcanCreER‐FOXO KO mice at 6 and 12 months of age (*n* = 6–8 mice per group). (d) Histopathological scores in the NP/AF and EP of lumbar IVD from AcanCreER^−/−^ and AcanCreER‐FOXO KO mice at 6 and 12 months of age (*n* = 6–8 mice per group). Values shown are mean ± *SD*. Statistical comparisons were assessed by an unpaired, two‐tailed *t*‐test after testing for equal variance using an *F*‐test. Values are mean ± *SD*. *p < 0.05

As mice with Col2a1Cre‐mediated FOXO deletion already developed spontaneous spine abnormalities before 4 months of age that were at least in part due to FOXO functions in postnatal growth and maturation, we used the AcanCreER model to analyze the role of FOXO in the maintenance of mature IVD. Four‐month‐old skeletally mature AcanCreER^−/−^; Foxo1^fl/fl^; Foxo3^fl/fl^; and Foxo4^fl/fl^ (AcanCreER^−/−^) and AcanCreER^+/−^; Foxo1^fl/fl^; Foxo3^fl/fl^; and Foxo4^fl/fl^ (AcanCreER‐FOXO KO) mice were injected with tamoxifen, and FOXO expression was assessed 2 weeks later by qPCR analysis (Supporting information Figure S5 in Appendix S1). Unlike the results in the Col2a1‐Cre model, the reduction in FOXO expression upon tamoxifen administration in the AcanCreER model was more marked in the NP than in the AF, consistent with higher aggrecan expression in NP than in AF (Anderson & Tannoury, 2005; Boos et al., 2002; Kadow et al., 2015). Histopathological analysis revealed no differences in cellularity or disk height between genotypes at 6 months of age (Figure 3a–c). However, at 12 months of age, there was a significant reduction in NP and EP cellularity and disk height in AcanCreER‐FOXO KO mice (Figure 3b). Clusters of NP cells surrounded by ECM were frequently found in the periphery of the NP of the mutant mice and this was associated with loss of NP/AF demarcation and abnormal organization of AF lamellae (Figure 3a). In contrast to Col2a1Cre‐FOXO KO mice, AcanCreER‐FOXO KO mice did not exhibit any abnormality in the vertebral growth plate, subchondral bone, or vertebral body (Supporting information Figure S6 in Appendix S1). Histopathological scores of the AcanCreER‐FOXO KO mice were significantly increased at 12 months for all IVD structures (Figure 3d). These findings suggest that FOXO are required for the maintenance of IVD integrity and that loss of FOXO in IVD in mature mice results in early onset IDD.

### 2.4 FOXO regulate expression of antioxidant and autophagic genes in NP

**Figure 4:**
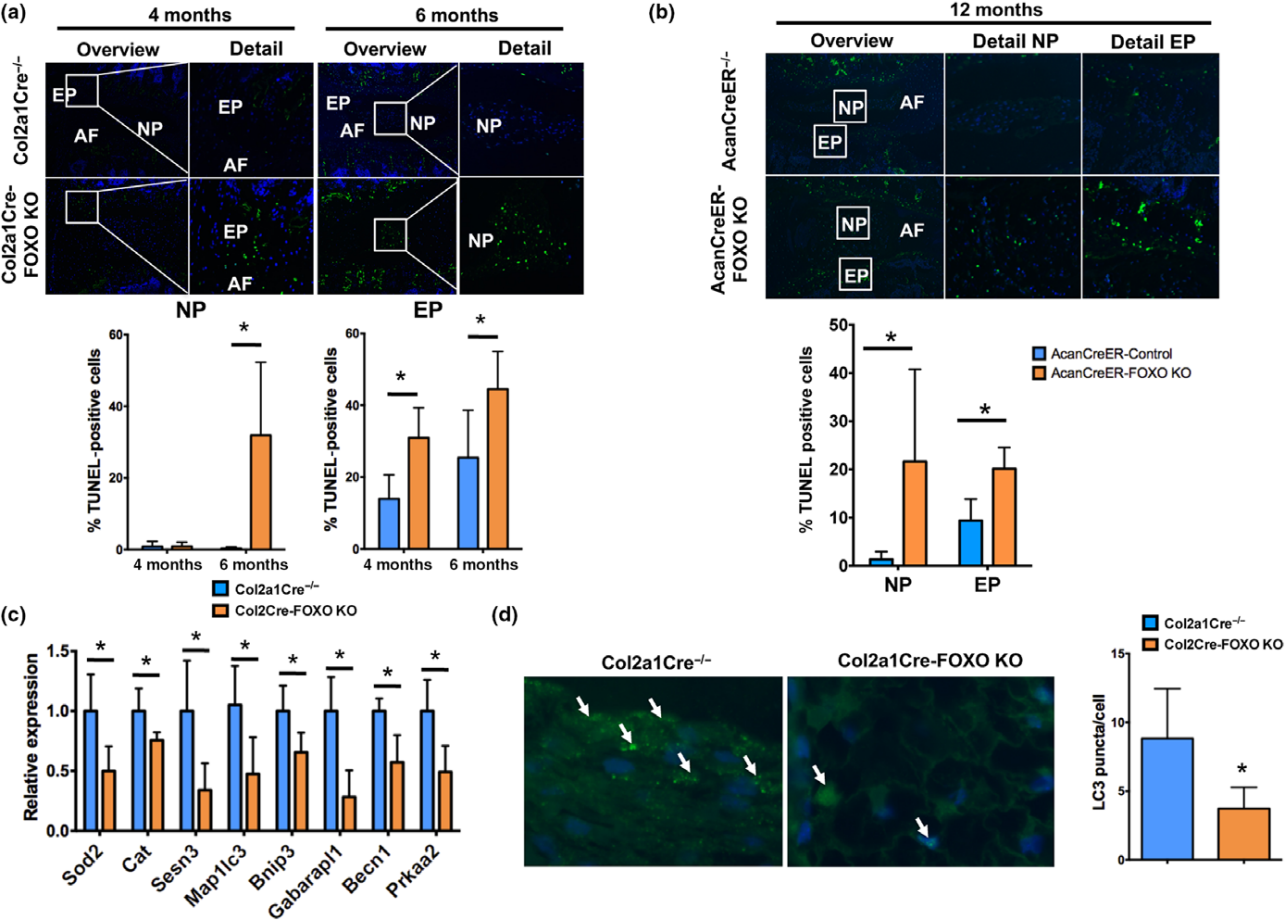
Reduced nucleus pulposus cell viability and impaired autophagy in FOXO‐deficient intervertebral disks. (a) Terminal deoxynucleotidyl transferase (TdT) dUTP Nick‐End Labeling (TUNEL) staining in lumbar intervertebral disks (IVD) isolated from Col2a1Cre^−/−^ and Col2a1Cre‐FOXO KO mice at 4 and 6 months of age (*n* = 5 mice per group). Lower panels show quantification of TUNEL‐positive cells in the NP and EP. No positive cells were observed in the AF. NP: nucleus pulposus; AF: annulus fibrosus; EP: endplate. Magnification bar = 100 µm. (b) TUNEL staining in lumbar IVD from AcanCreER^−/−^ and AcanCreER‐FOXO KO mice at 12 months of age (*n* = 5 mice per group). Lower panels show quantification of TUNEL‐positive cells in the NP and EP. (c) Gene expression analysis of homeostatic genes in NP from Col2a1Cre^−/−^ and Col2a1Cre‐FOXO KO mice at 2 months of age (*n* = 4 mice per group). (d) Immunofluorescence staining for LC3 in the NP of lumbar IVD isolated from Col2a1Cre^−/−^ and Col2a1Cre‐FOXO KO mice at 4 months of age (*n* = 5 mice per group) shows a decrease in immunostained cells in FOXO‐deficient NP cells. Right panel shows quantification of LC3 puncta per cell. Values shown are mean ± *SD*. Statistical comparisons were assessed by an unpaired, two‐tailed *t*‐test after testing for equal variance using an *F*‐test. Values are mean ± *SD*. *p < 0.05

Terminal deoxynucleotidyl transferase (TdT) dUTP Nick‐End Labeling (TUNEL) analysis revealed higher numbers of apoptotic cells in the EP of Col2a1Cre‐FOXO KO mice at 4 months and in the NP and EP at 6 months of age when compared with control littermates (Figure 4a). A similar increase in apoptotic cells was observed in the NP and AF of AcanCreER‐FOXO KO mice at 12 months of age (Figure 4b). Apoptotic cells were not detected in the AF of mice in any genotype or at any time point. Gene expression analysis showed a significant reduction in the antioxidant enzymes (*Sod2*,* Cat*,* Sesn3*) and autophagy‐related genes (*Map1lc3*,* Bnip3*,* Gabarapl1*,* Becn1*,* Prkaa2*) in the NP of 2‐month‐old FOXO mutant mice (Figure 4c). Moreover, the number of LC3 puncta, a marker of autophagosomes and autophagy activation (Mizushima & Yoshimori, 2007), was significantly decreased in the NP of Col2a1Cre‐FOXO KO mice at 4 months of age (Figure 4d).

**Figure 5:**
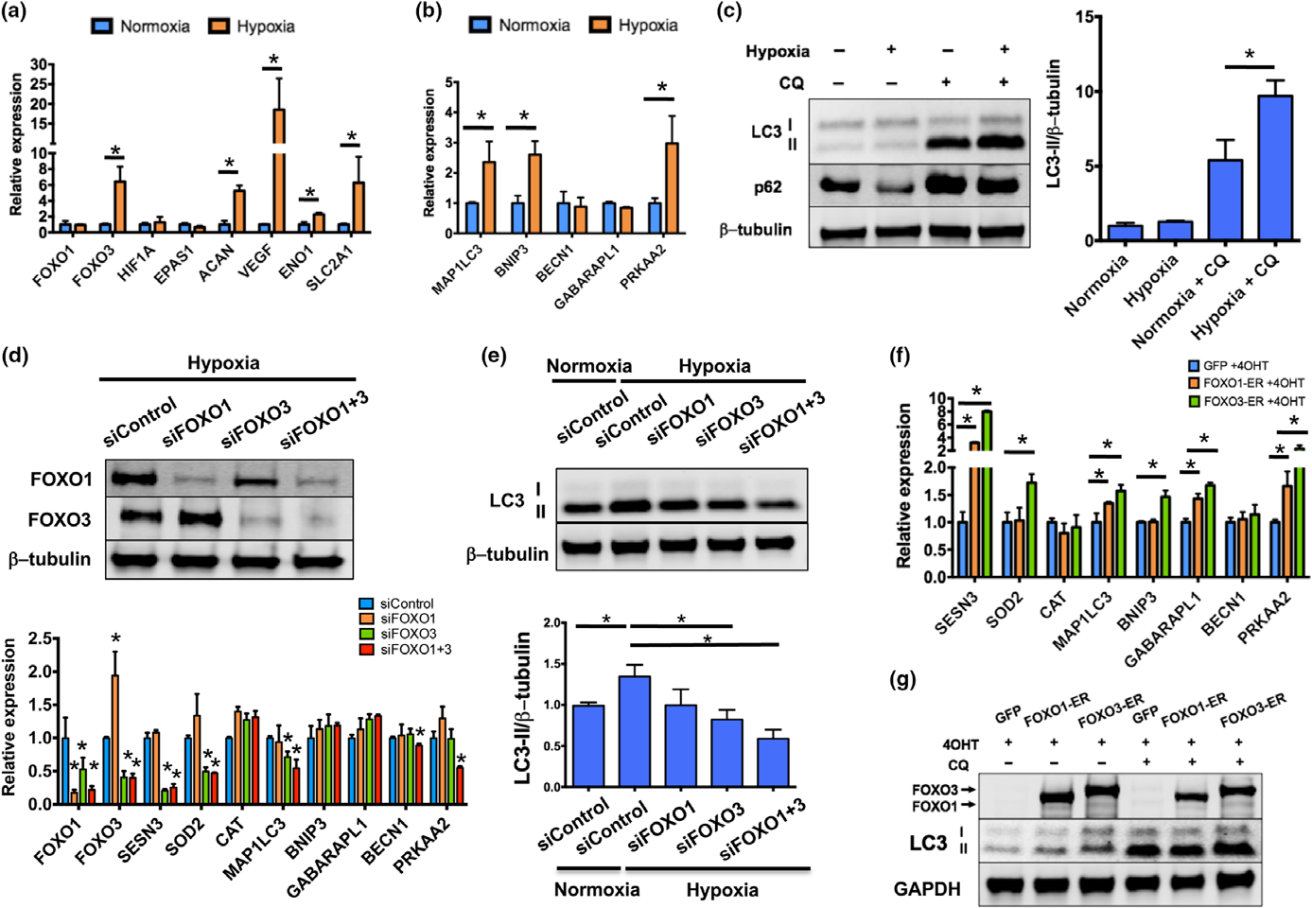
FOXO mediate hypoxia‐induced autophagy in human nucleus pulposus cells. (a,b) Gene expression analysis in human primary nucleus pulposus (NP) cells cultured under normoxia (20% oxygen) or hypoxia (1% oxygen) for 24 hr showing hypoxia‐induced activation of *FOXO3* and HIF1A signaling (a) and autophagic genes (b). (c) Western blot analysis of LC3‐I, LC3‐II, and p62 protein levels in human NP cells cultured in normoxia or hypoxia for 24 hr in the presence or absence of 25 µM chloroquine (CQ). Panel on the right shows densitometric quantification of LC3‐II and β‐tubulin. Values shown are mean ± *SD* of three different experiments. (d) Human NP cells were transfected with siRNA specific for FOXO1 (siFOXO1), FOXO3 (siFOXO3), or a combination of both (siFOXO1 + 3) and cultured in hypoxia for 24 hr. Upper panel shows Western blot analysis of FOXO1 and FOXO3 proteins confirming FOXO knockdown. Lower panel shows gene expression analysis of antioxidant and autophagic genes upon FOXO knockdown. (e) Western blot analysis of LC3 protein levels in human NP cells transfected with the indicated siRNA and cultured in normoxia or hypoxia for 24 hr in the presence of 25 µM CQ. Lower panel shows densitometric quantification of LC3‐II and β‐tubulin. (f) Gene expression analysis in human immortalized NP cells transfected with plasmids encoding for GFP, FOXO1‐ER, or FOXO3‐ER and treated with 1 µM 4OHT for 24 hr. (g) Western blot analysis of FOXO1, FOXO3, and LC3 protein levels in human NP cells transfected with plasmids encoding for GFP, FOXO1‐ER, or FOXO3‐ER and treated with 1 µM 4‐hydroxytamoxifen (4OHT) for 24 hr in the presence or absence of 25 µM CQ. Values shown are mean ± *SD*. Statistical comparisons were assessed by an unpaired, two‐tailed *t*‐test after testing for equal variance using an *F*‐test. *p < 0.05

NP cells reside in a unique microenvironment defined by low oxygen and low nutrients (Risbud, Schipani, & Shapiro, 2010). Adaptive mechanisms to this environment include activation of HIF1A (hypoxia‐inducible factor 1α subunit) signaling and increase in autophagic activity (Choi et al., 2016; Risbud et al., 2010). To determine whether FOXO expression and activity are modulated by hypoxia, human NP cells were cultured in 20% or 1% oxygen for 24 hr. Gene expression analysis showed increased mRNA levels of *ACAN* as well as of HIF1A targets *VEGF*,* SLC2A1*, and *ENO1 *under hypoxic conditions (Figure 5a). Notably, *FOXO3* but not *FOXO1* expression was upregulated by hypoxia (Figure 5a). This upregulation was not dependent of HIF1A as NP cells transfected with specific siRNA for *HIF1A *(siHIF1A) showed no differences in *FOXO3* levels (Supporting information Figure S7a in Appendix S1). In addition, hypoxia increased the expression of autophagic genes (*MAP1LC3*,* BNIP3*, and *PRKAA2*) and autophagic activity as evidenced by decreased protein levels of p62 and increased levels of lipidated LC3 (LC3‐II) (Figure 5b–c). In agreement with previous reports (Choi et al., 2016), this induction of autophagy by hypoxia was not regulated by HIF1A (Supporting information Figure S7b in Appendix S1).

Next, we sought to investigate whether FOXO regulate the expression of homeostatic genes in NP cells during hypoxic conditions. Human NP cells were transfected with siFOXO1, siFOXO3, or a combination of siFOXO1 + 3 and incubated in 1% oxygen for 24 hr. FOXO3 and FOXO1 + 3 knockdown significantly decreased expression of *SESN3*,* SOD2*, and *MAP1LC3*, whereas siFOXO1 + 3‐transfected cells also showed lower levels of *BECN1* and *PRKAA2* (Figure 5d). In addition, a significant reduction in LC3‐II levels was observed upon knockdown of FOXO3 and FOXO1 + 3 (Figure 5e). Conversely, ectopic overexpression of tamoxifen‐inducible forms of FOXO1 (FOXO1‐ER) or FOXO3 (FOXO3‐ER) in human immortalized NP cells (Sakai et al., 2004) increased mRNA levels of *SESN3*, *MAP1LC3*,* GABARAPL1*, and *PRKAA2* (Figure 5f). This transcriptional activation of homeostatic genes was likely a direct function of FOXO as overexpression of a FOXO3 mutant that lacks the DNA binding region (FOXO3‐∆DBD‐ER) (Tran et al., 2002) did not change gene expression (Supporting information Figure S8 in Appendix S1). In addition, LC3‐II levels were significantly elevated in cells overexpressing FOXO1 or FOXO3 (Figure 5g), indicating that FOXO is sufficient to stimulate autophagy in NP cells.

### 2.5 FOXO promote resistance to oxidative and inflammatory stress in NP cells

**Figure 6:**
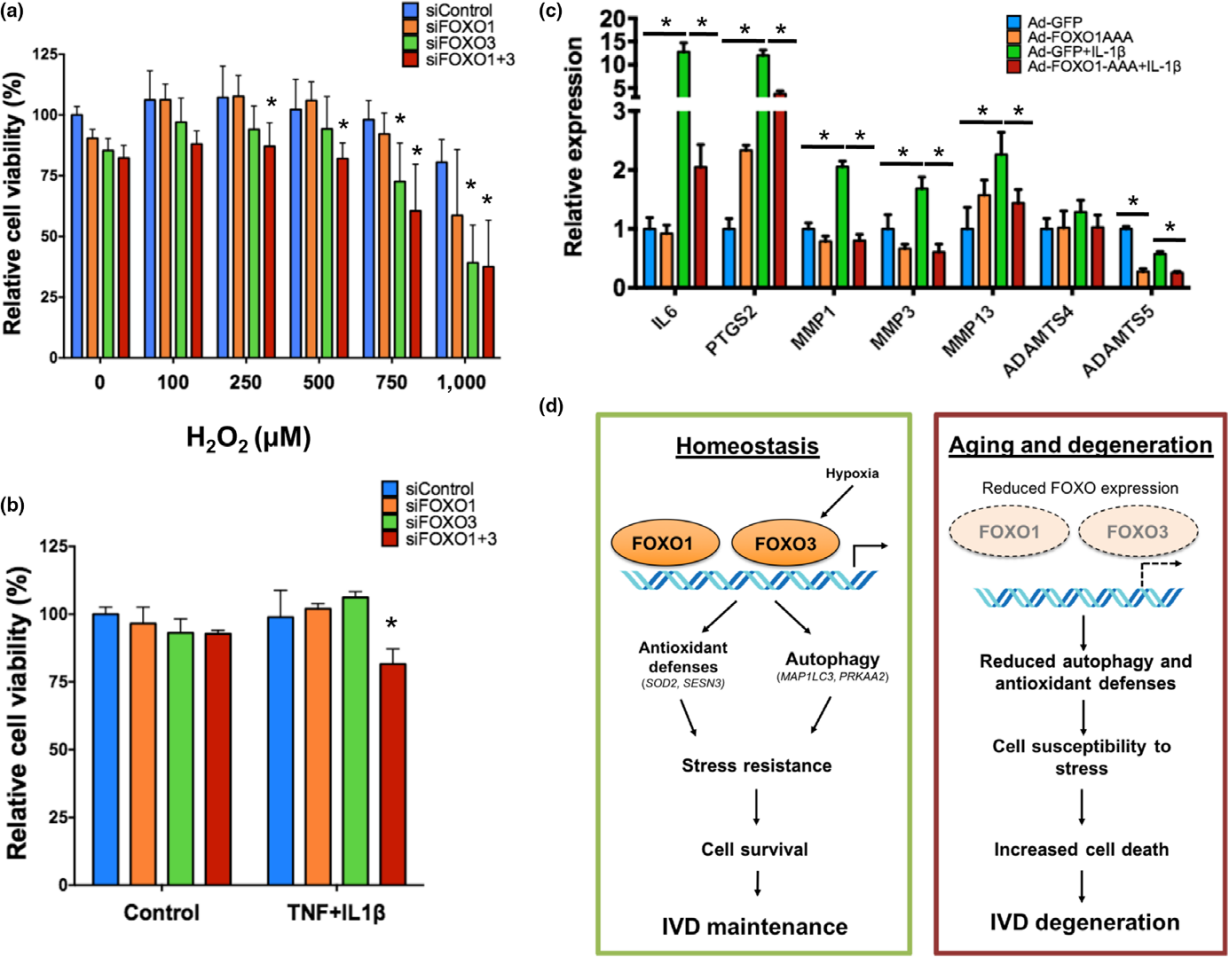
FOXO promote resistance to oxidative and inflammatory stress in human nucleus pulposus cells. (a, b) Cell viability analysis in human NP cells transfected with siRNA specific for FOXO1 (siFOXO1), FOXO3 (siFOXO3), or a combination of both (siFOXO1 + 3) and treated with different H_2_O_2_ concentrations (a) or TNF (10 ng/ml) and IL‐1β (10 ng/ml) (b) for 24 hr. (c) Cells from normal human NP were transduced with adenovirus encoding green fluorescent protein (GFP) or a constitutively active FOXO1 mutant (FOXO1‐AAA) and stimulated with IL‐1β (1 ng/ml). RNA was isolated after 6 hr for qPCR analysis. Values shown are mean ± *SD* of three different experiments performed in duplicate. Statistical comparisons were assessed by an unpaired, two‐tailed *t*‐test after testing for equal variance using an *F*‐test. *p < 0.05. (d) Schematic representation of FOXO mechanisms in regulating intervertebral disk (IVD) maturation and homeostasis. NP: nucleus pulposus; AF: annulus fibrosus; EP: endplate

To investigate whether a decrease in FOXO would compromise NP cell viability under stress conditions, human NP cells were transfected with siControl, siFOXO1, siFOXO3, or siFOXO1 + 3 and treated with increasing concentrations of H_2_O_2_ or with a cytokine cocktail (10 ng/ml of TNF‐α and 10 ng/ml of IL‐1β) in serum‐free media for 24 hr. Simultaneous knockdown of FOXO1 and FOXO3 resulted in a significant decrease in cell viability upon H_2_O_2_ or TNF‐α or IL‐1β stimulation (Figure 6a, b). In addition, at higher H_2_O_2 _concentrations, cell viability was significantly lower in siFOXO3‐treated cells, but not in cells with FOXO1 knockdown, suggesting that FOXO3 is the main isoform responsible for oxidative stress resistance in NP cells.

To examine the potential therapeutic benefit of targeting FOXO to preserve IVD homeostasis, human NP cells were transduced with adenovirus encoding for GFP (Ad‐GFP) or constitutively active FOXO1 (Ad‐FOXO1‐AAA) and treated with IL‐1β for 6 hr. IL‐1β stimulation increased expression of IL6, PTGS2, ADAMTS5, MMP1, MMP3, and MMP13 (Figure 6c). Remarkably, FOXO1 suppressed all IL‐1β ‐induced genes and also significantly reduced the basal expression of ADAMTS5 (Figure 6c).

## 3 DISCUSSION

Chronic pain and disability due to IDD is one of the most common age‐associated conditions in humans (Dagenais et al., 2008; Manchikanti et al., 2014). Aging is a major risk factor for the initiation and progression of IDD (Miller et al., 1988), and given the increase in life expectancy worldwide (2015), it has become a priority to identify the causes and mechanisms of IDD to reveal new therapeutic targets and approaches. Previous studies have proven useful in demonstrating the importance of changes in the expression of genes encoding structural components of ECM and growth factor signaling mediators in the pathophysiology of IDD (reviewed in Vo et al., 2013; Daly, Ghosh, Jenkin, Oehme, & Goldschlager, 2016). However, the precise molecular events that lead to cell dysfunction and potentially initiate the degenerative cascade during aging remain unknown. To our knowledge, this study provides the first evidence of specific transcription factors that regulate IVD aging and homeostasis. The focus on FOXO was based on their critical role in cellular homeostasis and aging (Eijkelenboom & Burgering, 2013; Webb & Brunet, 2014) and on our previous findings that the expression of FOXO is reduced in degenerated human IVD and during mouse spine aging (Alvarez‐Garcia, Matsuzaki, Olmer, Masuda, et al., 2017). Here, we report novel functions of FOXO in maintaining IVD integrity during aging by promoting essential homeostatic mechanisms in IVD cells, including autophagy, adaptation to the hypoxic environment, and protection against oxidative and inflammatory stresses.

Our data using the Col2a1Cre mouse model showed that loss of FOXO resulted in NP and EP cell death and tissue degeneration, suggesting a central role of FOXO in IVD homeostasis. However, these degenerative changes were also associated with profound abnormalities in growth and maturation of IVD and vertebrae with FOXO1 being the predominant isoform responsible for these changes as suggested by our isoform‐specific KO mouse models. As these developmental abnormalities could negatively impact the health of the IVD, we used the AcanCreER model to delete FOXO in skeletally mature mice. AcanCreER‐FOXO KO mice exhibited degenerative features that closely resembled the histological phenotype found in age‐related IDD characterized by a reduction in NP and EP cellularity, loss of NP/AF demarcation, and ossification of the EP (Alvarez‐Garcia, Matsuzaki, Olmer, Masuda, et al., 2017; Boos et al., 2002; Tam et al., 2017), but had no changes in the vertebral bodies. In addition, mice with conditional deletion of FOXO3 did not exhibit any IVD defects in postnatal growth or maturation but showed early onset of IDD with aging also characterized by a marked reduction in NP and EP cellularity, further supporting a critical role of FOXO in promoting mature IVD homeostasis by directly regulating cell survival.

Loss of NP cellularity is one of the earliest and most important events in IDD that is hypothesized to drive the progression of the disease (Anderson & Tannoury, 2005; Ding, Shao, & Xiong, 2013; Kadow et al., 2015; Vergroesen et al., 2015). NP cells are unique in their ability to adapt to their physiological environment, defined by low‐oxygen and nutrient levels (Bartels, Fairbank, Winlove, & Urban, 1998). In a recent study, Choi et al. (2016) showed that NP cells activate autophagy under hypoxic conditions through a noncanonical, HIF1A, and mTOR‐independent mechanism and that this activation of autophagy was required for long‐term NP cell survival. Here, we identified FOXO3 as a novel mediator of NP cell adaptation to hypoxia and demonstrated that FOXO3 and, to a lesser extent, FOXO1 regulate the expression of autophagy genes and activation in NP cells. Although a causal relationship between autophagy defects and IDD has not yet been established, our findings showed that FOXO function promotes NP cell survival under oxidative and inflammatory stresses, major drivers of NP cell apoptosis and aging‐related IDD (Nasto et al., 2013; Wang et al., 2013; Yang et al., 2014), and thus suggest that activation of FOXO in aged IVD could increase autophagy and protect NP cells from stress‐induced apoptosis. Further supporting this hypothesis, various studies have shown that autophagy activation can protect NP cells against different apoptotic insults (Jiang, Jin, Wang, Jiang, & Dong, 2014; Jiang, Zhang, et al., 2014).

In the present study, Col2a1Cre and AcanCreER drivers that are active in NP, AF, and EP (Henry et al., 2009; Jin et al., 2011) were chosen to delete FOXO in IVD to model the reduction of FOXO expression seen in all IVD compartments during mouse aging (Alvarez‐Garcia, Matsuzaki, Olmer, Masuda, et al., 2017). In addition to the severe degenerative changes in the NP, our data using both mouse models show that FOXO deletion led to cell loss and ossification in the cartilaginous EP. The EP has a pivotal role in regulating the nutritional status of the IVD and the age‐related decrease in EP permeability due to ossification has been suggested as a contributing factor in IDD (Bartels et al., 1998; Bernick & Cailliet, 1982; Wang, Battie, Boyd, & Videman, 2011). It is thus likely that reduced EP permeability secondary to EP calcification significantly contributes to the severe degeneration observed in FOXO‐deficient IVD. Importantly, the present findings imply that FOXO‐targeted therapies could preserve cell viability during aging and have beneficial effects on all IVD tissues.

The Col2a1Cre and AcanCreER drivers also cause FOXO deletion in other cartilaginous structures such as in the articular cartilage of the synovial joints and the growth plates (Henry et al., 2009; Zhu, Chen, Lichtler, O'Keefe, & Chen, 2008). We have recently reported the knee articular cartilage phenotype in these mouse models and demonstrated that FOXO are critical mediators of chondrocyte homeostasis by regulating PRG4 expression and maintaining the integrity of the cartilage superficial zone (Matsuzaki et al., 2018). We thus identified common and distinct molecular mechanisms that are regulated by FOXO in spine and synovial joints. The most important common feature of joints and spine in this regard is the aging and disease‐associated suppression of FOXO which could serve as a foundation to develop therapeutic interventions to prevent the onset of different aging‐related skeletal diseases such as IDD or osteoarthritis.

In conclusion, this study identified FOXO as essential regulators of IVD homeostasis during aging (Figure 6c). The protective effects of FOXO against oxidative stress, stimulation of autophagy and adaptation to hypoxia, and suppression of key catabolic mediators that contribute to IDD support the concept that the aging‐associated reduction in FOXO expression (Alvarez‐Garcia, Matsuzaki, Olmer, Masuda, et al., 2017) has important functional implications and may represent a central mechanism of IVD aging and risk factor for IDD. Therefore, maintaining or restoring FOXO expression during aging can be a therapeutic strategy to prevent or delay the onset the disease.

## 4 EXPERIMENTAL PROCEDURES

### 4.1 Animals

Col2a1‐Cre transgenic mice (Ovchinnikov, Deng, Ogunrinu, & Behringer, 2000) and AcanCreER knock‐in mice (Henry et al., 2009) on a C57BL6 background were obtained from the Jackson Laboratory (JAX#003,554, Bar Harbor, ME, USA). Foxo1^fl/fl^; Foxo3^fl/fl^; and Foxo4^fl/fl^ triple transgenic mice were obtained from Dr. R. DePinho (Paik et al., 2007). For IVD‐specific deletion of all three FOXO isoforms, Foxo1^fl/fl^; Foxo3^fl/fl^; and Foxo4^fl/fl^ mice were crossed with Col2a1‐Cre or AcanCreER mice. For deletion of single FOXO isoforms, Col2a1‐Cre mice were crossed with Foxo1^fl/fl^, Foxo3^fl/fl^, or Foxo4^fl/fl^ mice. Foxo1^fl/fl^; Foxo3^fl/fl^; and Foxo4^fl/fl^littermates not expressing Cre recombinase (Col2a1‐Cre^−/− ^or AcanCreER^−/−^) were used as controls. For FOXO deletion in skeletally mature mice, 4‐month‐old mice AcanCreER; Foxo1^fl/fl^; Foxo3^fl/fl^; and Foxo4^fl/fl^ mice were injected with tamoxifen (Sigma‐Aldrich, St. Louis, MO, USA) intraperitoneally at 1.5 mg/10 g body weight for five consecutive days.

### 4.2 Human IVD tissues

Normal human lumbar IVD samples were collected from five cadaveric donors (age 19–45 years). Macroscopic assessment was performed according to Thompson grading (Thompson et al., 1990) and confirmed the absence of any pathological changes. Additional details of the samples are listed in Supporting information Table S1 in Appendix S1.

### 4.3 Histological analyses

Lumbar spines were collected from FOXO mutant and control mice. Samples were fixed and decalcified as described previously (Alvarez‐Garcia, Matsuzaki, Olmer, Masuda, et al., 2017). The 4‐µm‐thick sagittal sections were stained with safranin O–fast green or picrosirius red staining for morphological analysis. Histological grading of NP/AF in mouse L4/L5 IVD was performed following the system described by Masuda et al. (2005), and degenerative changes in EP were graded according to a modification to the scoring system described by Boos et al. (2002). Briefly, the grading system in the NP and AF evaluated four different parameters: NP cellularity, border between NP and AF, matrix of the NP, and lamellar morphology in the AF. Each parameter was scored from 1 to 3 points, with 4 points (1 point in each category) representing normal histology and 12 points indicating severe degeneration. The scoring system for EP evaluated four parameters: cellularity (0–4 points), cartilage disorganization (0–4 points), cartilage continuity (0–4 points), and calcification/bone formation (0–4 points), for a combined score ranging from 0 to 16 points. At least six mice per experimental group were scored by two different observers blinded to the experimental conditions. Additional histomorphometric analysis was performed on images from L4/L5 IVD obtained under 20× magnification using imagej software. Disk height was calculated by averaging 20 parallel measurements of the distance between cranial and distal cartilage endplates. Cellularity of each IVD tissue and vertebral growth plate was calculated by dividing number of cells in each specific tissue by the area and was expressed in cell number/mm^2^. Vertebral diameter, number of trabeculae, and average trabecular thickness were measured in the distal subchondral bone at a distance of 50 µm of the growth plate.

### 4.4 Immunohistochemistry

Immunohistochemistry of lumbar spine sections was performed as described elsewhere (Alvarez‐Garcia, Matsuzaki, Olmer, Masuda, et al., 2017) using primary antibodies against collagen type X (1:10; X‐AC9; Developmental Studies Hybridoma Bank, Iowa City, IA,USA), RUNX2 (1:10; sc‐390351; Santa Cruz Biotechnology, Dallas, TX, USA), or IgG isotype control (Vector Laboratories, Burlingame, CA, USA). Five samples per experimental group were randomly selected for analysis.

### 4.5 Immunofluorescence

Lumbar spine sections were processed as described above and incubated overnight at 4°C with antibodies for collagen type II (1:100; II‐II6B3; Developmental Studies Hybridoma Bank), aggrecan (1:100; L0101; Assay Biotechnology, Fremont, CA, USA), or LC3 (1:200; PM036; MBL International, Woburn, MA, USA). Then, samples were incubated with secondary fluorescent antibodies (Alexa Fluor 488; 1:500; Thermo Fisher Scientific) and visualized under a fluorescence microscope. Five IVD samples per experimental group were analyzed. The number of LC3 puncta was quantified in 20 cells randomly selected in the NP, and data are expressed as average number of LC3 puncta per cell.

### 4.6 Cell proliferation and apoptosis

To evaluate the rate of cell proliferation, injections of 100 mg/kg of BrdU (Sigma‐Aldrich) were administered intraperitoneally to 3‐week‐old mice (*n* = 3 mice per group) for five consecutive days. Immunohistochemical detection of BrdU‐positive cells was performed using a primary antibody against BrdU (1:1,000, 66241‐1‐Ig; Proteintech, Rosemont, IL, USA) as indicated above. The number of positive cells was quantified in the NP of L4/L5 IVD under 20× magnification. Proliferation rate was expressed as the ratio of the total number of BrdU‐positive cells to the total number of NP cells in the L4/L5 IVD.

To assess the apoptotic cell death, TUNEL staining was performed using in situ cell death detection kit (Roche Applied Science, Indianapolis, IN, USA) in lumbar spine samples (*n* = 5 mice per group) and visualized under fluorescence microscopy. The number of positive cells was quantified in the NP and EP of L4/L5 IVD, and data were expressed as the ratio of TUNEL‐positive cells to the total number of DAPI‐positive cells in the L4/L5 disk.

### 4.7 Cell culture

Human NP cells were isolated following the protocol described by Sakai et al. (2012) and maintained in Dulbecco's modified Eagle's medium (DMEM) containing 10% calf serum (CS) at 37°C in 5% CO_2% _and 20% O_2_. Cells with less than three passages were used in all experiments. For hypoxia experiments, cells were cultured for 24 hr in a modular incubator chamber (MIC‐101; Billups‐Rothenberg Inc., Del Mar, CA, USA) filled with 5% CO_2% _and 1% O_2_. For the evaluation of autophagic activity, cells were incubated in the presence or absence of 25 µM chloroquine (CQ; Sigma‐Aldrich) for 24 hr. All experiments using human cells were performed at least three times in duplicate. A detailed description of gene knockdown, overexpression, and cell viability experiments is provided as Supporting information.

### 4.8 Gene expression analysis

Mouse lumbar spine samples were collected at 2 and 4 months of age. NP and AF from all lumbar IVD were resected separately for each mouse and homogenized in QIAzol Lysis Reagent (Qiagen, Valencia, CA, USA). RNA was isolated using Direct‐zol RNA miniprep kit (Zymo Research, Irvine, CA, USA). RNA quality was assessed by measuring the 260/280 absorbance ratio. All RNA samples used in this study had a 260/280 ratio > 1.8. Gene expression was measured by real‐time PCR using predesigned TaqMan gene expression assays (Thermo Fisher Scientific). At least four samples were analyzed in duplicate for each experimental condition.

### 4.9 Protein extraction and Western blotting

Protein extraction and Western blotting were performed as described previously (Alvarez‐Garcia, Matsuzaki, Olmer, Plate, et al., 2017). Specific antibodies used were FOXO1 (1:1,000; 2,880 T; Cell Signaling Technology, Danvers, MA, USA), FOXO3 (1:1,000; 2497S; Cell Signaling Technology), LC3 (1:1,000; 12,741 T; Cell Signaling Technology), p62 (SQSTM1, 8025S; 1:1,000; Cell Signaling Technology), HIF1A (1:1,000; 20960‐1‐AP; Proteintech) β‐tubulin (1:2,000; 66240‐1‐Ig; Proteintech), and GAPDH (1:5,000; AM4300; Thermo Fisher Scientific).

### 4.10 Statistics

All data are reported as the mean ± standard deviation (*SD*). All data were tested for normal distribution using the Kolmogorov–Smirnov test. Statistical comparisons between more than two groups were assessed by one‐way analysis of variance (ANOVA) followed by a post hoc Tukey's test. Comparisons between two groups were assessed by an unpaired, two‐tailed *t*‐test after testing for equal variance using an *F*‐test. All statistical analyses were performed using prism 6 software (GraphPad Software). *p*‐Values less than 0.05 were considered significant.

### 4.11 Study approval

All mouse studies were approved by the Scripps Institutional Animal Care and Use Committee and performed in accordance with ARRIVE guidelines and with attention to the standards reported by the NIH‐NINDS designed to enhance animal study transparency and reproducibility.

## CONFLICT OF INTEREST

The authors have declared that no conflict of interest exists.

## AUTHOR CONTRIBUTION

OA and ML designed the study; OA, TM, MO, KM, and SM performed experiments; OA, DS, KM, HA, and ML analyzed the data; OA and ML wrote and edited the manuscript. All authors reviewed and approved the final version of the manuscript.
