# Supplementary material for: FOXO are required for intervertebral disk homeostasis during aging and their deficiency promotes disk degeneration
Source: Aging Cell. 2018 Jul 2;17(5):e12800. doi: 10.1111/acel.12800 (PMC6156454; doi:10.1111/acel.12800)
Supplement: Supplementary file 1 [file ACEL-17-e12800-s001.docx]

**Supplemental data**

**Supplementary Experimental Procedures**

**FOXO knockdown in human NP cells**

Human NP cells were grown to confluence and transfected with small interfering RNA (siRNA) specific for FOXO1, FOXO3 or a scrambled control (Thermo Fischer Scientific, Waltham, MA USA) using Lipofectamine RNAiMAX (Thermo Fischer Scientific) in media supplemented with 10 CS for 48 hours.

**FOXO overexpression in human NP cells**

Human NP cells immortalized by transformation with the SV40 antigen (Sakai *et al.* 2004) were plated at 70% confluency and incubated with pcDNA3.1 plasmids encoding for green fluorescence protein (GFP), FOXO1-ER (Nogueira *et al.* 2008), FOXO3-ER (Tran *et al.* 2002) or FOXO3-∆DBD-ER (Tran *et al.* 2002) using Lipofectamine 3000 (Thermo Fischer Scientific) for 48 hours. Then, cells were treated with 1µM 4-hydroxytamoxifen (4OHT, Sigma-Aldrich) and RNA was collected after 24 hours. Primary NP cells were transduced with adenovirus encoding a constitutively active FOXO1 mutant (FOXO1-AAA) or GFP at 20 MOI in media containing 1% CS for 6 hours. Then, transduced cells were incubated for additional 36 hours in fresh media with 10% CS and stimulated with IL-1β for 6 hours (1 ng/ml; PeproTech).

**Cell viability**

Human NP cells were plated in 96-well plates at a density of 5000 cells/well and transfected with siRNA for FOXO1, FOXO3 or both as indicated above. After 24 hours, cells were treated with increasing concentrations of H_2_O_2_ or a combination of TNF (10 ng/ml; Peprotech) and IL-1β (10 ng/ml; PeproTech) for 24 hours in serum free media. Cell viability was assessed using RealTime-Glo™ MT Cell Viability Assay (Promega, Madison, WI, USA). Three independent experiments were performed in duplicate. Results are expressed as relative cell viability with respect to untreated cells transfected with siRNA control.

**Supplementary References**

Nogueira V, Park Y, Chen CC, Xu PZ, Chen ML, Tonic I, Unterman T, Hay N (2008). Akt determines replicative senescence and oxidative or oncogenic premature senescence and sensitizes cells to oxidative apoptosis. *Cancer Cell*. **14**, 458-470.

Sakai D, Mochida J, Yamamoto Y, Toh E, Iwashina T, Miyazaki T, Inokuchi S, Ando K, Hotta T (2004). Immortalization of human nucleus pulposus cells by a recombinant SV40 adenovirus vector: establishment of a novel cell line for the study of human nucleus pulposus cells. *Spine (Phila Pa 1976)*. **29**, 1515-1523.

Tran H, Brunet A, Grenier JM, Datta SR, Fornace AJ, Jr., DiStefano PS, Chiang LW, Greenberg ME (2002). DNA repair pathway stimulated by the forkhead transcription factor FOXO3a through the Gadd45 protein. *Science*. **296**, 530-534.

**Supplementary Tables**

**Supplementary Table 1:** Human intervertebral discs used in the study.

|  |  |  |  |
| --- | --- | --- | --- |
| **Disc Level** | **Morphologic Grade** | **Age** | **Gender** |
| L4-L5 | II | 19 | Female |
| L4-L5 | II | 40 | Male |
| L4-L5 | II | 45 | Female |
| L4-L5 | II | 45 | Female |
| L4-L5 | II | 49 | Male |

**Supplementary Figures**

**
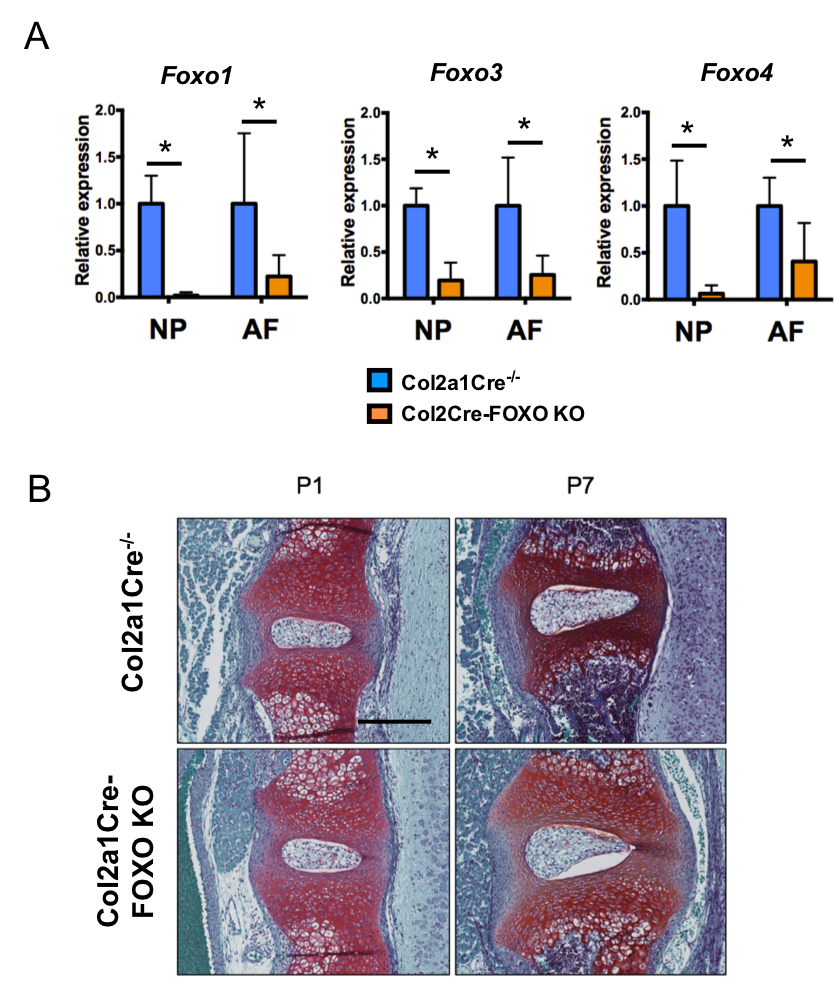
**

**Supplementary Figure 1 –** **Conditional FOXO deletion in IVD tissues.**

**A)** Analysis of FOXO mRNA levels in nucleus pulposus (NP) and annulus fibrosus (AF) samples of lumbar intervertebral discs isolated from Col2a1Cre^-/-^ and Col2a1Cre-FOXO triple KO (TKO) KO mice at 2 months of age (n=4 mice per group). Values shown are mean ± SD. Statistical comparisons were assessed by an unpaired, two-tailed T-test after testing for equal variance using an F-test. * = p < 0.05. **B)** Safranin-O staining of lumbar intervertebral discs isolated from Col2a1Cre^-/-^ and Col2a1Cre-FOXO TKO mice at 1 (P1) and 7 (P7) days postnatally. Magnification bar = 100 µm.

**
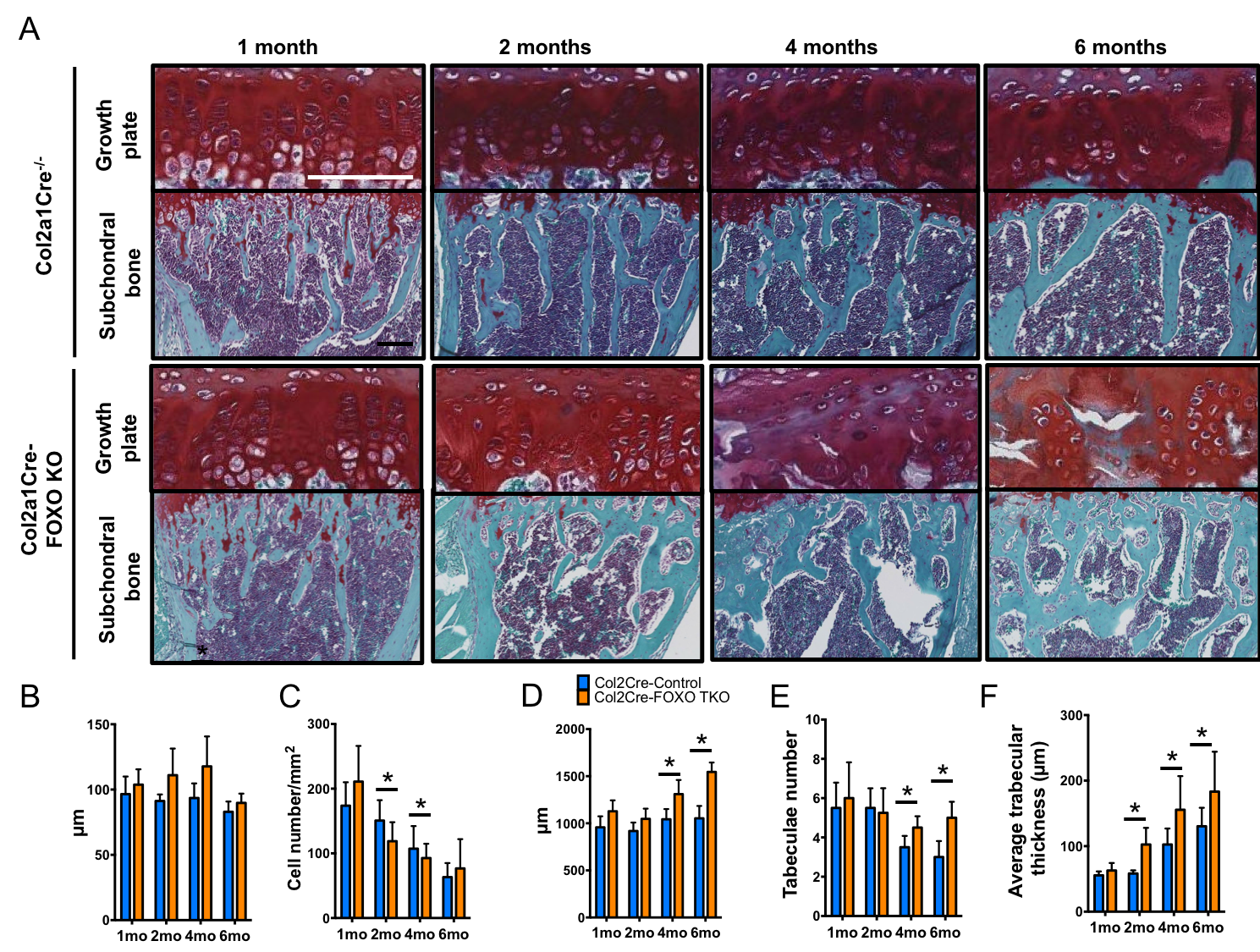
**

**Supplementary Figure 2 – FOXO deletion is associated with vertebral growth plate and subchondral bone abnormalities.**

**A)** Safranin O staining in lumbar IVD isolated from Col2a1Cre^-/-^ and Col2a1Cre-FOXO KO mice (FOXO Triple KO mice) at 1, 2, 4 and 6 months of age. **B-F)** Histomorphometrical analysis of vertebral growth plate thickness (**B**), vertebral growth plate cellularity (**C**), vertebral diameter (**D**), number of trabeculae (**E**), and average trabecular thickness (**F**) in lumbar IVD isolated from Col2a1Cre^-/-^ control and FOXO TKO mice at 1, 2, 4 and 6 months of age. Magnification bar = 100µm. Values shown are mean ± SD. Statistical comparisons were assessed by one-way analysis of variance (ANOVA) followed by a post-hoc Tukey test. * = p < 0.05.

**
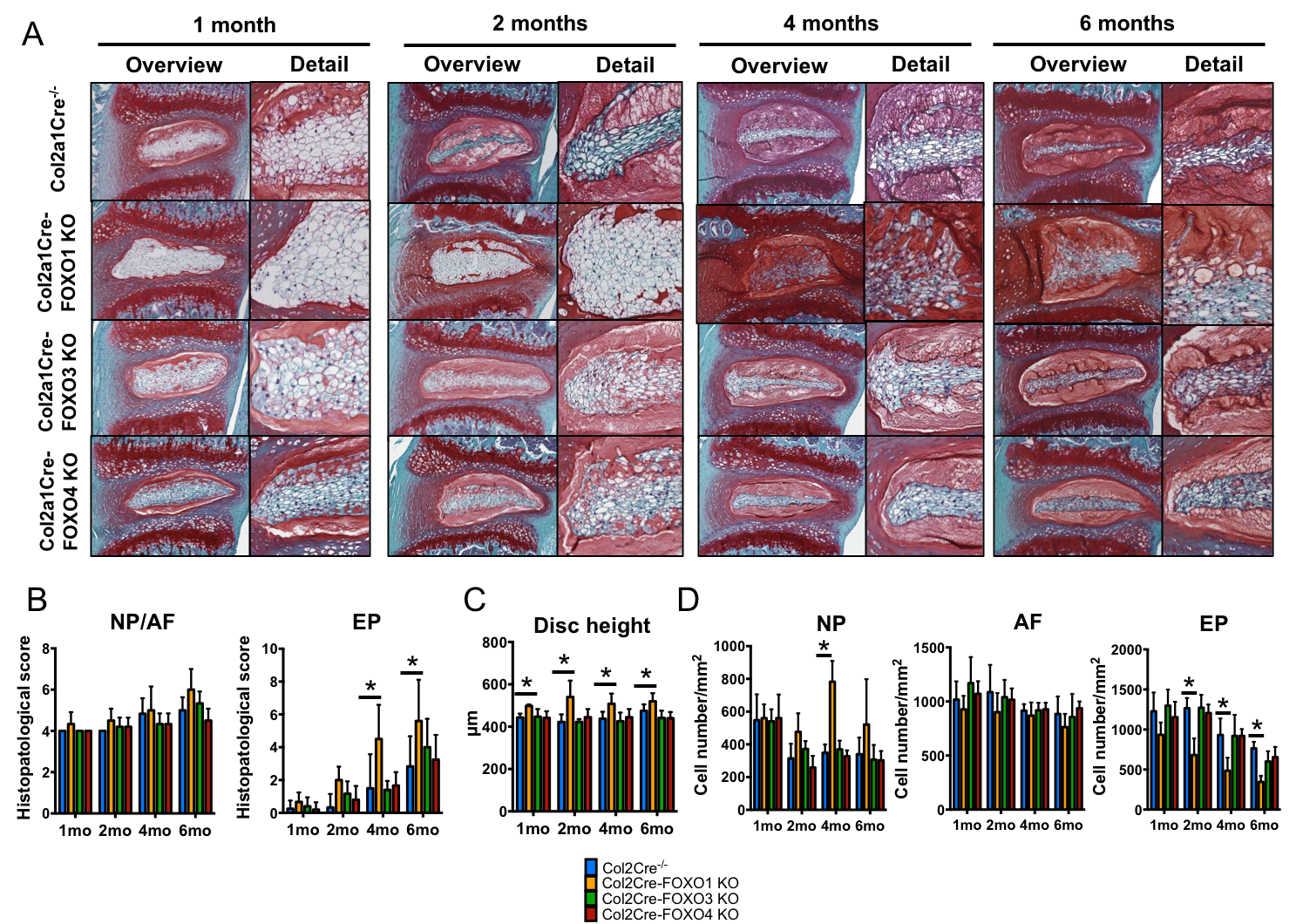
**

**Supplementary Figure 3 – Phenotypic differences in mice with conditional deletion of individual FOXO isoforms in intervertebral disc.**

Col2a1Cre^-/-^, Col2a1Cre-FOXO1 KO, Col2a1Cre-FOXO3 KO, and Col2a1Cre-FOXO4 KO mice at 1, 2, 4 and 6 months of age were analyzed (n=6-8 mice per group). NP = nucleus pulposus. AF = annulus fibrosus. EP = endplate. **A)** Safranin O staining in lumbar intervertebral disc (IVD) samples **B)** Histopathological scores in the nucleus NP/AF and EP of lumbar IVD samples. **C)** Measurement of disc height of lumbar IVD. **D)** Quantification of cellularity in the NP, AF, and EP of lumbar IVD). Magnification bar = 100µm. Values shown are mean ± SD. Statistical comparisons were assessed by one-way analysis of variance (ANOVA) followed by a post-hoc Tukey test. * = p < 0.05.

**
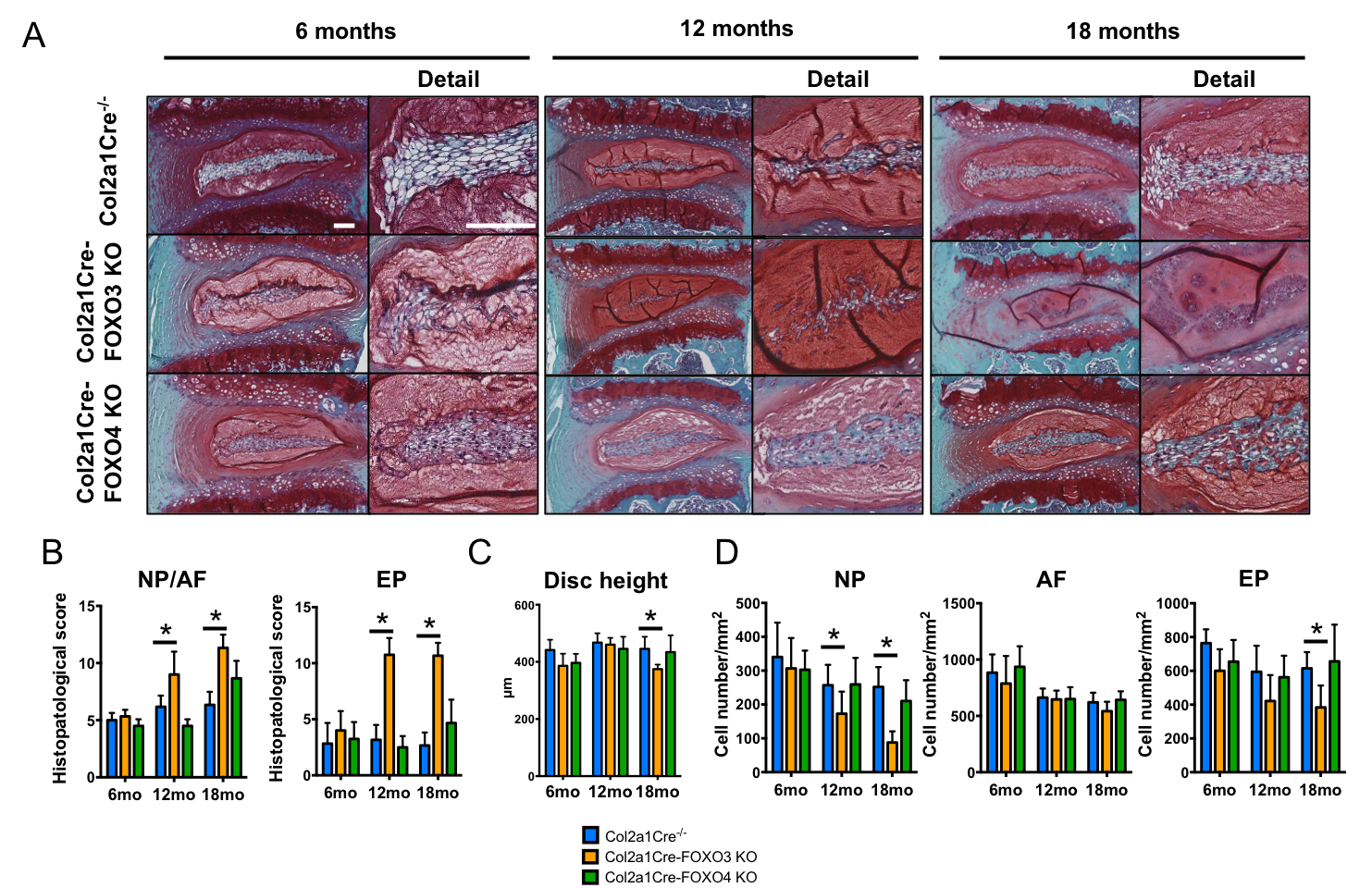
**

**Supplementary Figure 4 – Age-related intervertebral disc degeneration upon deletion of FOXO3.**

**A)** Safranin O staining in lumbar IVD isolated from Col2a1Cre^-/-^, Col2a1Cre-FOXO3 KO and Col2a1Cre-FOXO4 KO mice at 6, 12 and 18 months of age. **B)** Histopathological scores in NP/AF and EP of lumbar IVD samples from Col2Cre^-/-^, Col2a1Cre-FOXO3 KO and Col2a1Cre-FOXO4 KO mice at 6, 12 and 18 months of age. **C)** Disc height of lumbar IVD samples from Col2a1Cre^-/-^, Col2a1Cre-FOXO3 KO and Col2a1Cre-FOXO4 KO mice at 6, 12 and 18 months of age. **D)** Cellularity in the NP, AF, and EP of lumbar IVD samples from Col2a1Cre^-/-^, Col2a1Cre-FOXO3 KO and Col2a1Cre-FOXO4 KO mice at 6, 12 and 18 months of age. At least 6 mice per group were analyzed. Magnification bar = 100µm. Values shown are mean ± SD. Statistical comparisons were assessed by one-way analysis of variance (ANOVA) followed by a post-hoc Tukey test. * = p < 0.05.

**
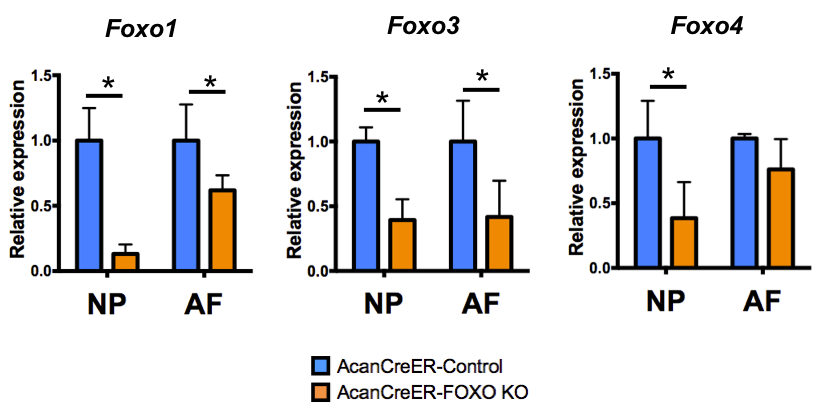
**

**Supplementary Figure 5 -** **FOXO gene expression in lumbar intervertebral discs of skeletally mature mice with conditional FOXO deletion.**

Analysis of FOXO mRNA levels in nucleus pulposus (NP) and annulus fibrosus (AF) samples isolated from AcanCreER^-/-^ and AcanCreER-FOXO KO mice 2 weeks after tamoxifen injection (n=4 mice per group). Values shown are mean ± SD. Statistical comparisons were assessed by an unpaired, two-tailed T-test after testing for equal variance using an F-test. * = p < 0.05.

**
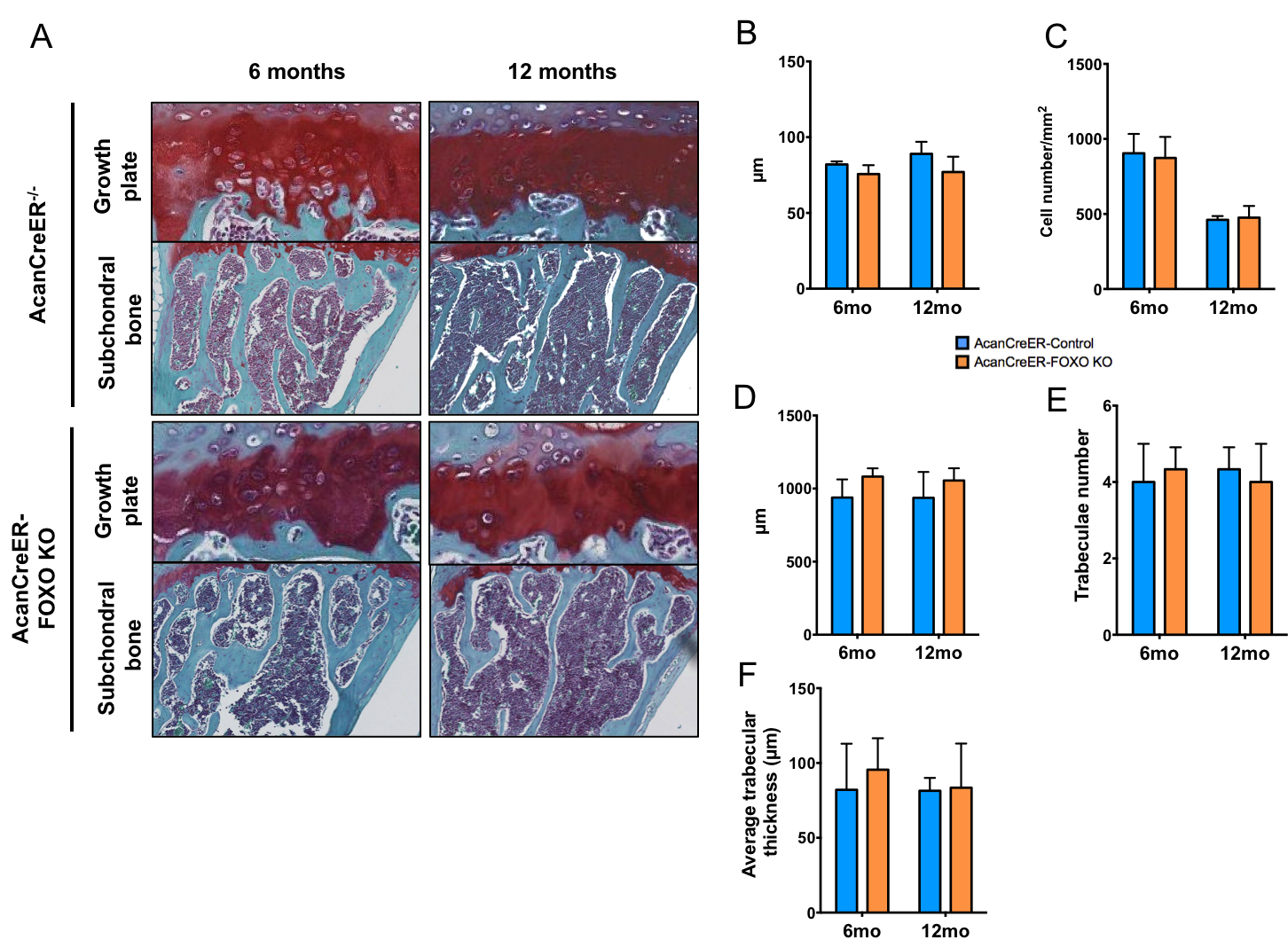
**

**Supplementary Figure 6 – Normal vertebral growth plate and subchondral bone histomorphology upon FOXO deletion in skeletally mature mice.**

**A)** Safranin O staining in lumbar IVD isolated from AcanCreER^-/-^ and AcanCreER-FOXO KO mice at 6 and 12 months of age. **B-F)** Histomorphometrical analysis of vertebral growth plate thickness (**B**), vertebral growth plate cellularity (**C**), vertebral diameter (**D**), number of trabeculae (**E**), and average trabecular thickness (**F**) in lumbar IVD isolated from AcanCreER^-/-^ and AcanCreER-FOXO KO mice at 6 and 12 months of age. Magnification bar = 100µm. Values shown are mean ± SD. Statistical comparisons were assessed by an unpaired, two-tailed T-test after testing for equal variance using an F-test. * = p < 0.05.

**
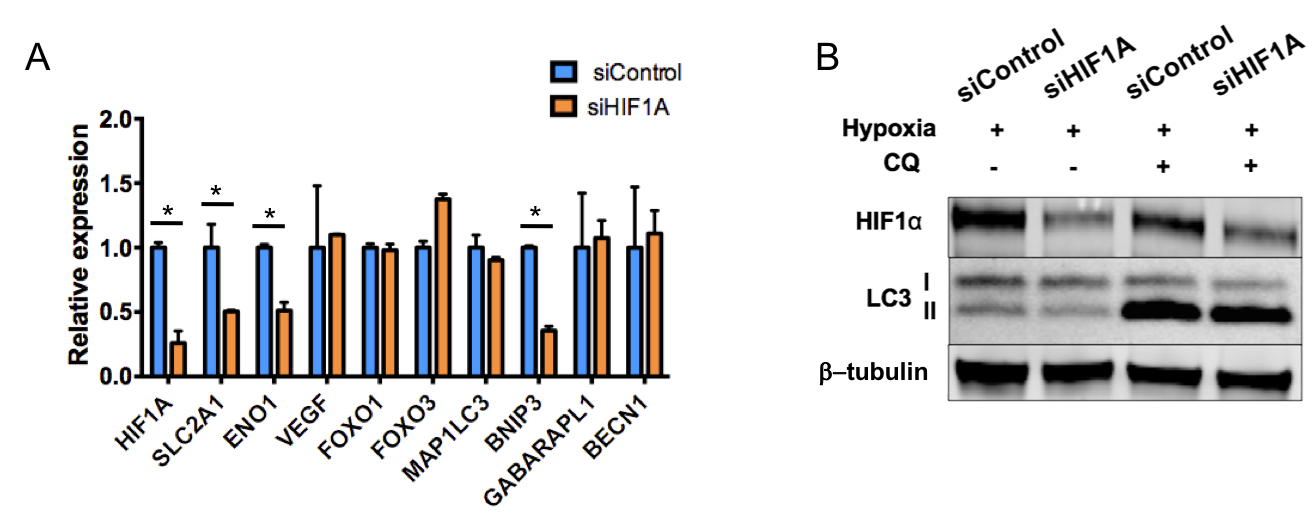
**

**Supplementary Figure 7 - FOXO expression and autophagy induction by hypoxia is not dependent on HIF1A.**

**A)** Gene expression analysis in human NP cells transfected with siRNA control (siControl) or specific for HIF1A (siHIF1A) for 48 hours under 1% oxygen. **B)** Western blot analysis of HIF1A and LC3 protein levels in human NP cells transfected with siControl or siHIF1A and cultured under 1% oxygen in the presence or absence of 25µM chloroquine (CQ). Values shown are mean ± SD. Statistical comparisons were assessed by an unpaired, two-tailed T-test after testing for equal variance using an F-test. * = p < 0.05.

**
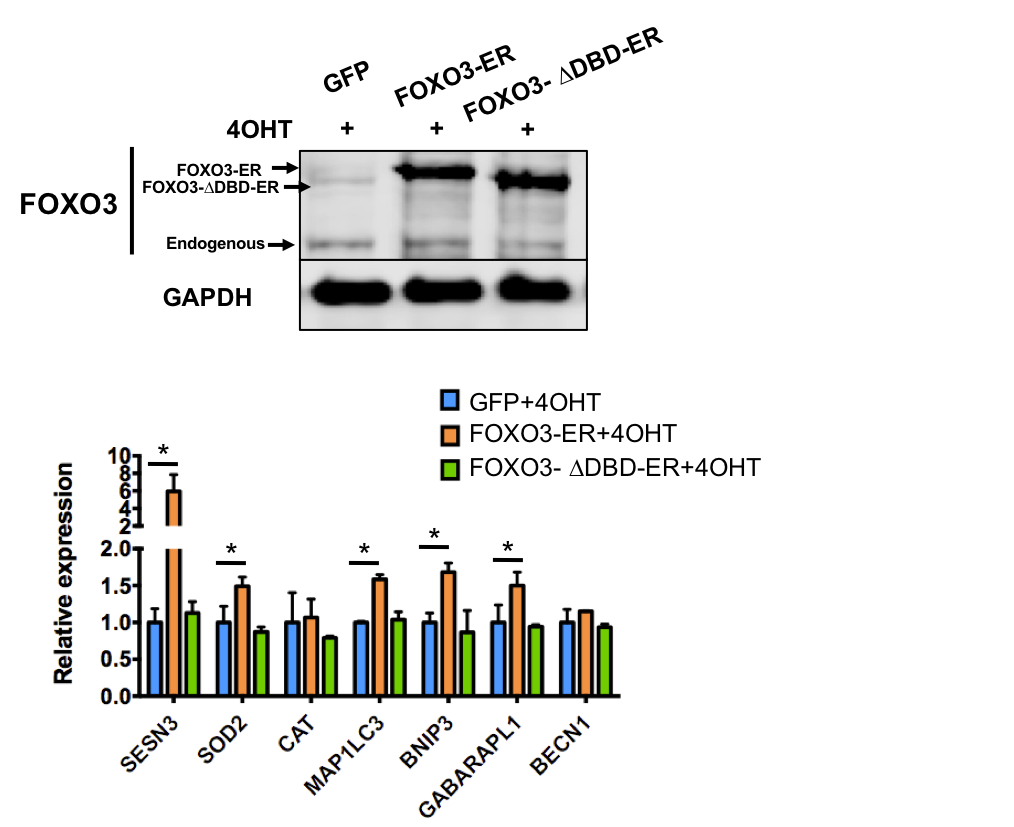
**

**Supplementary Figure 8 - DNA binding of FOXO3 is required for induction of homeostatic genes in human nucleus pulposus cells.**

Immortalized human nucleus pulposus (NP) cells were transfected with plasmids encoding for GFP, FOXO3-ER and a FOXO3 mutant that lacks the DNA binding domain (FOXO3-∆DBD-ER) and treated with 1µM 4-hydroxytamoxifen (4OHT) for 24 hours. Upper panel shows western blot analysis of FOXO proteins. Lower panel shows gene expression analysis of antioxidant and autophagic genes in NP cells with GFP, FOXO3-ER or FOXO3-∆DBD-ER overexpression. Values shown are mean ± SD. Statistical comparisons were assessed by one-way analysis of variance (ANOVA) followed by a post-hoc Tukey test. * = p < 0.05.
